# Supplementary material for: Content-rich biological network constructed by mining PubMed abstracts
Source: BMC Bioinformatics. 2004 Oct 8;5:147. doi: 10.1186/1471-2105-5-147 (PMC528731; doi:10.1186/1471-2105-5-147)
Supplement: Additional File 2 — The original results of the above study (non-essential files are deleted to keep the file size under the limit set by BMC bioinformatics). [file 1471-2105-5-147-S2.bz2 › chilibotAdditionalFile2/dip10/527ID8663349E10832/html/CBX5_LBR.html]

 


 **CBX5** and **LBR** 
  
Found 8 abstracts in PubMed, retrieved 8.  
 

 What does Google say? 
 PDF only 
| .edu only 

---

**Interactive relationship** (e.g. stimulation, inhibition, etc)

**Neutral relationship**- The modular domain organization of HP1  [ **CBX5** ]  type proteins and  **LBR**  can explain some of the diverse protein protein interactions at the chromatin lamina membrane interface of the nuclear envelope.  Ref: 9169472 J Biol Chem, 1997
- The interactions of the  **LBR**  domains with an HP1  [ **CBX5** ]  type protein were also analyzed by the yeast two hybrid and in vitro binding assays, which showed that a portion of the second globular domain is necessary for binding.  Ref: 9169472 J Biol Chem, 1997
- **LBR**  fusion proteins bound to HP1  [ **CBX5** ]  proteins synthesized by in vitro translation and present in cell lysates.  Ref: 8663349 J Biol Chem, 1996
- Domain specific interactions of human HP1  [ **CBX5** ]  type chromodomain proteins and inner nuclear membrane protein  **LBR** .  Ref: 9169472 J Biol Chem, 1997
- We show here that the chromo shadow domain mediates the self associations of HP1  [ **CBX5** ]  type proteins and is also necessary for binding to  **LBR**  both in vitro and in the yeast two hybrid assay.  Ref: 9169472 J Biol Chem, 1997
- These include interactions between nuclear lamins and core histones, Lamina Associated Polypeptide 2 LAP2, and the Barrier to Autointegration Factor BAF and interactions between lamin B receptor  [ **LBR** ]   **LBR**  and the chromodomain protein HP1  [ **CBX5** ] .  Ref: 10651245 Crit Rev Eukaryot Gene Expr, 1999
- Using purified components and in vitro assays, we now demonstrate that HP1  [ **CBX5** ]  forms a quaternary complex with the inner nuclear membrane protein  **LBR**  and a sub set of core histones.  Ref: 11571267 EMBO Rep, 2001
- HP1  [ **CBX5** ]  type chromodomain proteins self associate as well as interact with the inner nuclear membrane protein  **LBR**  lamin B receptor  [ **LBR** ]  and transcriptional coactivators TIF1alpha and TIF1beta.  Ref: 9169472 J Biol Chem, 1997
- In this report, we have reconstituted and characterized the HP1  [ **CBX5** ]  KAP 1 interaction using purified proteins and have compared KAP 1 to three other known HP1  [ **CBX5** ]  binding proteins SP100, lamin B receptor  [ **LBR** ]   **LBR** , and the p150 subunit from chromatin assembly factor CAF 1 p150.  Ref: 10938122 Mol Cell Biol, 2000
- In a yeast two hybrid screen, the nucleoplasmic domain of lamin B receptor  [ **LBR** ]   **LBR** , an integral protein of the inner nuclear membrane, associated with two human polypeptides homologous to Drosophila HP1  [ **CBX5** ] , a heterochromatin protein involved in position effect variegation.  Ref: 8663349 J Biol Chem, 1996
- The lamins and the lamin binding proteins lamina associated polypeptide LAP 2beta and lamin B receptor  [ **LBR** ]   **LBR**  have been described to bind to DNA or to interact with chromatin via histones, BAF 1, and HP1  [ **CBX5** ]  chromodomain proteins, respectively, and may provide anchorage sites for chromatin fibers at the nuclear periphery.  Ref: 10651242 Crit Rev Eukaryot Gene Expr, 1999

**Non-interactive relationship** (e.g. studied together, co-existance, homology, etc.)

- This complex involves histone H3 H4 oligomers, which mediate binding of  **LBR**  to HP1  [ **CBX5** ]  and cross link these two proteins that do not interact directly with each other.  Ref: 11571267 EMBO Rep, 2001
- **LBR**  can interact independently with lamin B and a chromodomain protein, Hp1  [ **CBX5** ] , and appears to be a central player in targeting nuclear membranes to chromatin.  Ref: 9442884 Annu Rev Cell Dev Biol, 1997
- Antibodies against  **LBR**  also co immunoprecipitated HP1  [ **CBX5** ]  proteins from cell extracts.  Ref: 8663349 J Biol Chem, 1996
- Consistent with previous observations, HP1  [ **CBX5** ]  and  **LBR**  binding to core histones is strongly inhibited when H3 H4 are modified by recombinant CREB binding protein, revealing a new mechanism for anchoring domains of under acetylated chromatin to the inner nuclear membrane.  Ref: 11571267 EMBO Rep, 2001
